# Digital Health Innovations to Catalyze the Transition to Value-Based Health Care

**DOI:** 10.2196/57385

**Published:** 2025-01-20

**Authors:** Lan Zhang, Christopher Bullen, Jinsong Chen

**Affiliations:** 1 Department of Public Administration, Law School, Hangzhou City University, Hangzhou, China; 2 National Institute for Health Innovation, University of Auckland, Auckland, New Zealand; 3 School of Public Affairs, Zhejiang University, Hangzhou, China

**Keywords:** digital health, value-based health care, VBHC, patient-reported outcome measures, PROM, digital transformation, health care innovation, patient-centric care, health technology, patient-reported outcome, PRO, outcome measure, telehealth, telemedicine, eHealth, personalized, customized, engagement, patient-centered care, standardization, implementation

## Abstract

The health care industry is currently going through a transformation due to the integration of technologies and the shift toward value-based health care (VBHC). This article explores how digital health solutions play a role in advancing VBHC, highlighting both the challenges and opportunities associated with adopting these technologies. Digital health, which includes mobile health, wearable devices, telehealth, and personalized medicine, shows promise in improving diagnostic accuracy, treatment options, and overall health outcomes. The article delves into the concept of transformation in health care by emphasizing its potential to reform care delivery through data communication, patient engagement, and operational efficiency. Moreover, it examines the principles of VBHC, with a focus on patient outcomes, and emphasizes how digital platforms play a role in treatment among tertiary hospitals by using patient-reported outcome measures. The article discusses challenges that come with implementing VBHC, such as stakeholder engagement and standardization of patient-reported outcome measures. It also highlights the role played by health innovators in facilitating the transition toward VBHC models. Through real-life case examples, this article illustrates how digital platforms have had an impact on efficiencies, patient outcomes, and empowerment. In conclusion, it envisions directions for solutions in VBHC by emphasizing the need for interoperability, standardization, and collaborative efforts among stakeholders to fully realize the potential of digital transformation in health care. This research highlights the impact of digital health in creating a health care system that focuses on providing high-quality, efficient, and patient-centered care.

## Introduction

In the last few decades, the health care landscape has undergone changes due to the rapid advancement and integration of technology. This technological progress has transformed how health care is managed, both for individuals and on a scale, by providing insights into patient health and experiences through digital recording and data collection methods.

Nowadays these advancements in technology are collectively known as “digital health.” According to the Food and Drug Administration, digital health encompasses a range of technologies, including mobile health (mHealth), health information technology, wearable devices, telehealth, telemedicine, and personalized medicine [1]. The adoption of health technologies is increasingly common in the health care industry, offering prospects for improved diagnostic accuracy, treatment options, and overall health outcomes [2].

These innovations in health are driving a shift toward a value-based approach [3]. This places patients at the center of care delivery and marks a departure from health care models. However, navigating the complex digital health ecosystem poses challenges for transformations in health care.

## Digital Transformation

Digital transformation refers to “a process that aims to improve an entity by triggering significant changes to its properties through combinations of information, computing, communication, and connectivity technologies” [4]. Digital transformation is reforming the modern-day health care system. Propelled by the COVID-19 pandemic, we have witnessed a surge of technology innovation to assure continuity in care delivery [5].

However, in the context of the health industry, digital transformation not only refers to managing clinical data flows and using advanced technologies but also a wider philosophical framework of business and operation transformation via digital innovations and technologies to increase stakeholder satisfaction. Its aim goes beyond digitizing the organizational environment to facilitate the use of data and leverage the value of data insights for improving clinical governance and performance evaluation, supporting clinical decision-making and operation efficiency, as well as ensuring the highly efficient utilization of health resources [5]. Ultimately, the process of digital transformation for the health care system needs to consider how to increase the quality of care and patient satisfaction via enhancing clinical data communication and patient engagement [6].

The digital transformation of the health care industry has leveraged a range of digital solutions in each part of the patient journey, including patient assessment, treatment, and management. These digital innovations arose out of the need for treatment and patient care, but toward multidimensional well-being and multi-disciplinary health support. The digital transformation enables health care professionals and other stakeholders in the health system to accomplish value-based health care (VBHC) more efficiently and sustainably [7].

## Value-Based Health Care

VBHC, defined as a health care activity that highlights value as patient-centric outcomes over the cost invested to treat patients [8,9], is one of the dominant trends in the global health industry [3,10,11]. Due to the high costs and growing inefficiency of health care in many nations, as well as the growing agreement that the existing health care paradigm is essentially unsustainable [12,13], advocating and implementing a VBHC model has become a strategic imperative for many health systems [14,15].

What is value? A definition proposed by the European Commission broadens the interpretation of “value” from a single variable based solely on monetary value in the context of cost-effectiveness to a more inclusive concept. The proposed definition of “value” includes the following four components [16]: (1) allocative “value”: equitable distribution of resources across all patient groups; (2) technical “value”: achievement of the best possible outcomes with available resources; (3) personal “value”: appropriate care to achieve patients’ personal goals; and (4) societal “value“: contribution of health care to social participation and connectedness.

A system-level model and framework may be necessary to determine “value” within the many components connected by complex processes inside a health care system [17].

Under VBHC, the focus shifts from volumes (the number of physician visits, hospitalizations, procedures, and tests) to outcomes (the patients’ prioritized health outcomes, such as an increase in functional independence and quality of life [18]. With a focus on the quality of patient care, the aim is to reduce the time spent in the health care sectors, avoid the development of chronic diseases, and minimize deterioration of medical conditions [18]. VBHC empowers patients and enables caregivers to provide better care at a lower cost, resulting in benefits for all stakeholders: patients, providers, payers, suppliers, and society [18].

## Patient-Reported Outcome Measures

An important element of VBHC is measuring outcomes that matter to patients, including their symptoms, functionality, and their satisfaction with their health care experience. This led to the introduction and adoption of patient-centered outcomes assessments, which take into account aspects of cure and survival, as well as recovery and quality of life retained [19,20]. A patient-reported outcome (PRO) is “an outcome reported directly by patients themselves and not interpreted by an observer.” PROs bring the “patient’s voice” to the care journey and identify conditions or symptoms that matter the most to a patient’s daily life and quality of life [21].

To measure PROs, instruments known as patient-reported outcome measures (PROMs) have been developed. These instruments are typically self-reported questionnaires that measure symptom burden, functional status, health-related quality of life, and health-related behaviors such as anxiety [22]. They are generally categorized into 2 groups: general health-related quality of life PROMs and disease-specific PROMs [22]. General PROMs focus on general well-being, mental state, and quality of life across a range of medical conditions [22], whereas disease-specific PROMs capture a combination of symptom severity and impact resulting from a specific condition or treatment [23].

PROMs are an essential part of VBHC because they measure what matters most to patients, and this drives the health care system to take a more holistic and comprehensive view of the physical, mental, and social impacts of illness. Research has found PROMs can be used in different ways to contribute to VBHC, such as enhancing patients’ and clinicians’ communication for identifying treatment plans; quantifying and monitoring symptoms and illness impact on life; reflecting patients’ preference on treatment and care; evaluating the quality of care and improvement of health; facilitating patient adherence and satisfaction with care; and promoting informed utilization of health resources and services [24].

Traditionally, PROMs were administered and distributed on paper throughout a patient’s care journey, requiring manual efforts to distribute, collect, and calculate patient scores. This can be a time-consuming and inefficient process, with additional administrative burden for clinical teams. However, web-based software, platforms, and apps are now being used for end-to-end management of PROMs, from automation of distributing, capturing, analyzing, and reporting these data. Features include automatic scoring of PROM data, interpretive dashboards and reports for clinicians, alerts to notify clinical teams of patients’ acute needs for symptom management, integration with electronic health records, and notification reminders tailored for patients [25].

One example of a fully digital PROMs system is the Symptom Tracking and Reporting (STAR) system used to assess a patient’s functional recovery after radical prostatectomy at Memorial Sloan-Kettering Cancer Center [26]. The web-based system is used to collect PROMs on urinary function, sexual function, bowel function, and overall quality of life. Patients are automatically emailed a unique link with access to a web-based portal for symptom reporting. Clinicians and patients can view numerical and graphical summaries of patients’ reported PROM scores, with prediction models of a patient’s future functional status. Tell Us, a web-based system designed for patients with advanced cancer in palliative care, has similar functionalities to STAR, but with the additional ability to trigger real-time alerts to clinical teams when intervention is necessary. The field is growing rapidly [5,27].

Previous studies had confirmed the effect of PROMs on patients in tertiary hospitals [28,29]. A study in a tertiary care center among patients with septoplasty and functional endoscopic sinus surgery found that due to the high response rates, PROMs that use mobile digital patient engagement platforms could effectively monitor postoperative outcomes [30]. Another study in a tertiary care center specialized in skull base diseases also showed that administering digital PROMs seems appropriate for evaluating health-related quality of life in skull base disorders [31].

## Research Gaps and Study Aims

In conclusion, the integration of digital transformation, VBHC, and PROMs had been discussed in the previous study [32-34]. However, few studies had ever investigated the specific role and impact of digital health solutions in driving VBHC [5], as well as specific clinical efficiency gains and resource savings from the adoption of digital health technologies [35] and the specific ways and effects of how digital health solutions could be used to personalize treatment [36,37].

Thus, this article aimed to explain the role that digital health solutions played in advancing VBHC. It examined the challenges that came with adopting these technologies and highlighted the work of health startups that were committed to assisting health care organizations in their shift toward value-based care models.

## Key Challenges

Although digital transformation facilitates the transition of traditional health models toward VBHC, implementation and adaptation are also needed from the many different stakeholders [38]. A shift in the overall cultural and behavioral environment requires a transition in clinical governance, management, and policy making, which in turn needs the joint contributions of health care professionals, payers, policy makers, caregivers, and, crucially, patients.

One of the key challenges of implementing VBHC in a health system is to facilitate and incentivize stakeholders to commit to capturing and reporting PROs along with clinical outcomes. Taking insights generated from PROs into decision-making processes (eg, care plan development, selection of treatments or therapies) unleashes the power and value of PROs and other patient-generated health data (PGHD), such as objective data captured by wearable devices. However, the applications of PROs and other PGHD into routine patient care will lead to a disruption of the health care governance and clinical practice.

Despite a growing consensus on the utility of PROs in clinical care, most of their applications remain in research or academic initiatives [39]. One major barrier to integrating PROs into clinical practice is the lack of standardization of how PROs are measured [40]. Measurement of PROs can range from unstructured patient diaries to structured clinically validated questionnaires, such as PROMs. More sophisticated questions, such as when to disseminate PROMs to patients, how often to send these PROMs, and what data analysis should be done with the data, are usually raised once the appropriate PROMs are identified for a target patient group. These questions highlight the importance of standardizing the method of PROMs selection and implementation.

Even if there are incentives and standardized PROMs, leadership, resources, and support are needed to encourage current health systems and physicians to change. Introducing an additional workflow of PRO data collection and application may present significant increases in workload for health care providers. This is where a professionally developed and validated digital solution can play its role in the process of digital transformation of the health system toward VBHC. Not only can digital solutions reduce the burden of collecting and using PRO data, but they should also support health care providers in their routine workflow (eg, enabling patient screening, follow-up care, support of self-management, etc), which will further incentivize health care providers to use the platform.

## Digital Health Approach

Digital health innovators are at the forefront of advocating for the adoption of VBHC, particularly in addressing the challenges of real-world implementation. Their mission is to assist health care organizations globally in transitioning to a VBHC model by providing tools that promote a patient-centered care approach.

Typically, digital solutions involve cloud-based Software as a Service digital platforms with automated and streamlined digital care pathways, reducing manual and costly processes. Digital care pathways use digital technologies to coordinate the monitoring, engagement, and support of patients throughout their care continuum, consolidating these processes into a unified system that collects, analyzes, and manages PGHD, including PROs and wearable or medical device data. They also deliver educational information to patients and providers.

Moving beyond traditional electronic data capture systems and generic customer relationship management platforms, digital platforms can seamlessly combine clinical and non-clinical data through integration with electronic medical records and clinical workflows. Thus, with a fully configurable, disease and device-agnostic platform, health professionals are equipped with a single, patient-centric system for managing communication, data collection, and analysis, providing real-time actionable information and insights to inform and improve patient care and management. The digital care pathway enhanced access to patient information and accelerated the provision of early specialist care [41]. Previous study had confirmed that digital transmural care pathway was feasible and beneficial in daily clinical care for patients with lung cancer [42].

For each medical condition and treatment, there are four broad phases in developing value-based digital care pathways: (1) defining schedules, (2) content creation, (3) automated patient engagement, and (4) insight generation. In developing new digital care pathways, they assist health care organizations in identifying areas of digitization that best benefit individual organizations and align with best practice recommendations, such as the frameworks set up by the International Consortium for Health Outcomes Measurement (ICHOM). Every touchpoint within a patient’s care journey is digitally transformed and automated from their admission to discharge, including communication sent out to patients, the type and frequency of health data collected, the educational material delivered, and reports to be generated. For each digital interaction, a patient’s health data is automatically captured and presented with actionable insights, ensuring providers have access to the right information for delivering high-quality care.

All 4 key components of value (allocative, technical, personal, and societal) can be enhanced through adopting digital health platforms [18]. The digital platform helps in the following ways.

The first is by improving data collection. Digital innovations transform the way data is collected, shared, and analyzed, significantly enhancing clinical decision-making, improving quality of care, and reducing health care costs [43]. The digital platform enables efficient collection of patient-generated data (eg, PROMs) through automation, thus overcoming barriers like time constraints and resource limitations. This leads to a broader scope of data collection beyond clinical data, reducing the time spent administering assessments. A systematic review found that electronic platforms simplified the implementation of PROMs in the lung cancer daily clinic [44].

The second is by enhancing clinical outcomes. To improve clinical health outcomes, both health care providers and patients need access to specific tools for the collection and analysis of patient data: assessment forms, wearables, or medical devices. These specialized tools are being used more and more to identify health risks and support diagnosis, treatment, and monitoring of both health and disease [45]. In this way, health care providers gain a holistic understanding of each patient’s unique health status and needs, leading to better-informed diagnosis and treatment decisions.

The third is by improving the performance and standardization of PROMs. Addressing the challenge of standardization in value-based care is crucial. The digital platform aids in implementing a structured and standardized approach to collecting and processing PROMs. A digital platform can allow for detailed measurement of recovery by regularly collecting PROMs, which can be used clinically to support recovery. Using a digital platform makes it possible to automate PROM data collection and characterize recovery across multiple domains, and this approach is likely to be implementable in clinical settings [29]. By adopting digital and automated condition-specific care pathways, health care providers can obtain high-quality data that drive personalization of patient diagnostic and treatment plans and achieve cross-organizational benchmarking for performance and quality of care improvement.

## Case Example: Implementation of Digital Platform for PROMs

Patients with lung cancer undergoing chemotherapy commonly experience a range of symptoms that vary in severity, with some indicative of critical oncological emergencies. To monitor patients’ symptoms and progress, traditional care processes can take up to 30 minutes, which includes having a nurse practitioner call patients for a weekly check-in, manually noting and transferring this information to an electronic medical record, and then working through required interventions. However, this is a time- and resource-intensive process, inefficient for the management of increasing workloads and patients.

Based on the following reasons, a hospital in Brisbane was selected to work with. First, a tertiary hospital in Brisbane, Australia, was selected as the case study site based on the actual needs and achievements of the hospital in the application of digital health platforms. There was a high demand for the management of postchemotherapy side effects in patients with lung cancer at this hospital, and it was already practically applying the digital platform for monitoring and intervention, which provides a realistic basis for the study. Second, the hospital’s challenges in managing postchemotherapy side effects, such as inefficiencies in traditional methods that result in high time and resource costs, make the hospital’s real-world problem consistent with the study’s goals on how to improve health care efficiencies and patient outcomes through digital health innovations based on PROMs. Working with the hospital, the authors used a digital health platform to monitor and address postchemotherapy side effects in patients with lung cancer. The platform was used to configure a digital care pathway that enabled the automation of the collection and analysis of 12 key posttreatment chemotherapy-related symptoms using PROMs, with real-time alerts when oncological emergencies were reported. This initiative has had positive impacts for both clinical teams and patients: improved clinical efficiencies, patient outcomes, and patient empowerment.

Capturing patient responses allowed clinical teams to effectively analyze patient outcomes, contributing to improved and personalized treatment decisions. Clinical teams have noted significant time and cost savings, with an average reduction of 10 posttreatment phone calls, spanning 20 minutes per call, to identify patients requiring clinical interventions [46]. Improved operational efficiency has allowed the oncology team to handle an increasing workload while maximizing patient outcomes.

Additionally, real-time data on patient conditions improve clinical decision-making and reduce unnecessary hospitalization and travel costs for patients. Clinical teams have noted that employment of the digital health platform has allowed the effective identification of patients suitable for treatments without hospital consultations compared to those requiring a doctor’s review. By including patients’ voices in the decision-making process, it empowers patients to actively engage in their treatment plans and feel confident in reporting their outcomes using the digital service.

To conclude, at the hospital in Brisbane, the digital platform enabled clinical process optimization by automating the monitoring and handling of chemotherapy side effects. The use of this digital platform demonstrated that patient care could be significantly more efficient and effective by collecting and analyzing patient data in real time. The digital platform not only reduced the reliance on traditional phone checks but also provided a real-time alert system that quickly identified patients in need of urgent care. This approach highlighted the practical application of digital health solutions in VBHC and their contribution to improving patient outcomes. Meanwhile, the case hospital reduced phone check-in time per patient through the digital platform, reducing the number of calls to be handled from an average of 10 to 0. In addition, the clinical team was able to more efficiently identify and prioritize patients requiring urgent intervention. These improvements not only increased efficiency but also saved significant time and resources, allowing the hospital to handle a larger patient load. This illustrated how digital health tools could help hospitals improve clinical efficiency despite limited resources. Furthermore, the digital platform enabled the hospital’s oncology team to provide more personalized care through real-time data analytics that allowed them to adjust treatment plans based on each patient’s specific symptoms. This customized approach to treatment helped optimize outcomes for each patient and improve overall quality of care. It illustrated how digital platforms could support VBHC through a personalized approach, enhancing the precision and effectiveness of patient care.

## Contribution

This paper could make several important contributions to the field of VBHC and the knowledge base of digital transformation in health care. First, it enhanced understanding of digital health solutions by analyzing the roles of various digital health technologies, including mHealth, wearable devices, telehealth, and personalized medicine. This study clarified how these innovations contributed to the goals of VBHC. It provided a nuanced discussion on how these technologies could improve diagnostic accuracy, expand treatment options, and enhance overall health outcomes. Second, this research identified key challenges in the implementation of VBHC, such as the need for stakeholder engagement and the standardization of PROMs. It provided a detailed discussion of these challenges and proposed potential solutions, thereby offering practical guidance for overcoming barriers to VBHC adoption. Third, this study provided empirical evidence of digital platforms through real-life case studies. This study demonstrated the tangible impacts of digital platforms on health care efficiencies, patient outcomes, and patient empowerment, and the case example offered concrete evidence of how digital health solutions could facilitate the transition to VBHC models and improved care delivery.

Overall, this paper contributed valuable insights into how digital transformation and VBHC could be effectively integrated to create a health care system that prioritizes high-quality, efficient, and patient-centered care.

## Future Directions

Digital solutions have played a growing role in the accomplishment of VBHC. In the near future, digital solutions with strong interoperability and accreditation from academic or standardization development bodies (eg, ICHOM) will have their competitive advantages. With the increasing number of digital solutions and systems to be used by a health system, interoperability plays a vital role in determining how easily a digital solution can work across different systems. Working closely with academic or standardization development bodies will ensure digital solutions are more usable and adaptable by health care providers, with measures developed or codeveloped by providers and validated in real-world settings. Due to the lack of standardized regulatory and coding standards with the provision of PROMs, revision and development of policies to support standards for PROMs usage and interoperability need to be better developed for the successful adoption of value-based models. With better regulation and policies in place, this would encourage more funding opportunities for value-based initiatives. While it has been a complex health care process, we have seen the accelerated growth and adaptation enabled by the COVID-19 pandemic [47], with many more untapped needs to be addressed.

## Conclusions

The paradigm shift toward VBHC represents not merely a shift in health care delivery metrics but also a fundamental transformation in how health care value is perceived and measured. Central to this transformation is the role of digital technology, which has become a cornerstone in the global pursuit of VBHC.

Digital transformation in health care involves far more than just the adoption of new technologies. It encompasses the standardization of measures to ensure consistency and comparability across different systems and regions. This standardization is crucial for accurately assessing and comparing health care outcomes, which is a fundamental aspect of VBHC. Additionally, innovation in data applications plays a pivotal role. The ability to effectively collect, analyze, and use vast amounts of health data can drive improvements in patient care, enhance operational efficiency, and inform policy decisions.

Engagement with stakeholders is another critical element in this transition. VBHC requires the active participation of all parties involved in health care, including providers, patients, payers, policy makers, and technology developers. Each stakeholder brings unique insights and expertise, contributing to a more holistic approach to health care reform. Collaborative efforts are essential in addressing the challenges and leveraging the opportunities presented by digital transformation.

The execution of digital transformation in health care offers numerous opportunities for stakeholders to work together toward a common goal. This collaboration can lead to the development of innovative solutions, the optimization of resource allocation, and the delivery of care that truly meets the needs and expectations of patients. Moreover, it can ensure that the benefits of VBHC are realized broadly, leading to tangible improvements in health care outcomes, patient satisfaction, and system sustainability.

In conclusion, the journey toward VBHC is complex and challenging, yet it is filled with immense potential. By embracing digital transformation and fostering collaboration among all stakeholders, the health care sector can achieve a system that not only values quality over quantity but also delivers care that is equitable, efficient, and truly patient-centered. The future of health care lies in harnessing the power of digital innovation to create a system where value and quality are at the forefront of every decision and action.

## Abbreviations

ICHOM: International Consortium for Health Outcomes Measurement
mHealth: mobile health
PGHD: patient-generated health data
PRO: patient-report outcome
PROM: patient-reported outcome measure
STAR: Symptom Tracking and Reporting
VBHC: value-based health care
